# Long‐term efficacy of meaning‐centered group psychotherapy for cancer survivors: 2‐Year follow‐up results of a randomized controlled trial

**DOI:** 10.1002/pon.5323

**Published:** 2020-02-06

**Authors:** Karen Holtmaat, Nadia van der Spek, Birgit Lissenberg‐Witte, William Breitbart, Pim Cuijpers, Irma Verdonck‐de Leeuw

**Affiliations:** ^1^ Department of Clinical, Neuro‐ and Developmental Psychology, Amsterdam Public Health Research Institute (APH) Vrije Universiteit Amsterdam Amsterdam The Netherlands; ^2^ Cancer Center Amsterdam (CCA) Amsterdam Public Health Research Institute (APH) Amsterdam The Netherlands; ^3^ IDC Center for Psycho‐Oncology Care OLVG Hospital Amsterdam The Netherlands; ^4^ Department of Epidemiology and Biostatistics, Amsterdam UMC Vrije Universiteit Amsterdam Amsterdam The Netherlands; ^5^ Department of Psychiatry and Behavioral Sciences Memorial Sloan Kettering Cancer Center New York New York; ^6^ Department of Otolaryngology/Head and Neck Surgery Amsterdam UMC Amsterdam The Netherland

**Keywords:** cancer, cancer survivors, depression, follow‐up studies, group psychotherapy, intention‐to‐treat analysis, oncology, outcome assessment, psychological adaptation, psychological stress

## Abstract

**Objective:**

Meaning‐centered group psychotherapy for cancer survivors (MCGP‐CS) is an effective intervention to improve personal meaning, psychological well‐being, and depressive symptoms until 6 months after the intervention. In this study, the long‐term effects of MCGP‐CS (i.e., at 1‐ and 2‐year follow‐up) on meaning, psychological well‐being and posttraumatic growth were assessed, in comparison to supportive group psychotherapy (SGP) and care as usual (CAU).

**Methods:**

Cancer survivors (n = 170) were randomized into MCGP‐CS, SGP, or CAU. Assessments were scheduled at baseline, 1 week, 3 months, 6 months, 1 year, and 2 years postintervention. Outcome measures were the Personal Meaning Profile, Ryff's Scales of Psychological Well‐Being (SPWB), the Posttraumatic Growth Inventory, and their subscales. Linear mixed models (LMM) were used and results were both reported on an intention‐to‐treat (ITT) basis, as well as for intervention completers only.

**Results:**

LMM and post hoc analyses with Bonferroni correction revealed that MCGP‐CS participants reported more improvement on positive relations (subscale of SPWB) than CAU participants of 2‐year postintervention (ITT analysis, Cohen's *d* = .82). Completers also reported more personal growth (subscale of SPWB) after MCGP‐CS than after SGP 1‐year postintervention (Cohen's *d* = .94). No long‐term effects were found on the other outcome measures.

**Conclusions:**

In the 2 years after MCGP‐CS, the short‐term significant effects on personal meaning and most positive effects related to psychological well‐being faded. However, MCGP‐CS had a long‐term positive effect on positive relations with others and on survivors' sense of personal growth.

**Trial registration:**

Netherlands Trial Register: NTR3571

## 1 BACKGROUND

Many cancer survivors encounter physical hindrances and are confronted with psychosocial and existential problems, also years after curative treatment is completed.^1^, ^2^ There is growing evidence that meaning‐focused coping is a viable way to successfully adjust to the aftermath of cancer,^2^ especially if meaning can be made from the cancer experience.^3^, ^4^ Breitbart and colleagues developed meaning‐centered group psychotherapy (MCGP) to improve psychological well‐being in patients with advanced cancer.^5^, ^6^ This intervention is grounded in the work of the psychiatrist Viktor Frankl,^7^ founder of logotherapy (ie, meaning therapy). MCGP was adapted for cancer survivors (MCGP‐CS) by Van der Spek et al.^8^ MCGP‐CS focuses on enhancing a sense of meaning in life by addressing issues like: how to carry on in life despite limitations, choosing one's attitude toward suffering, and discussing sources of meaning in life.

There is evidence that MCGP and MCGP‐CS are effective in enhancing a sense of meaning, psychological well‐being, and reducing depressive symptoms.^5^, ^8^, ^9^, ^10^ MCGP‐CS is likely to be cost‐effective as well.^11^ In a randomized controlled trial (RCT) among advanced cancer patients, MCGP was more effective than supportive group psychotherapy (SGP) in improving quality of life, spiritual well‐being, and reducing depression and hopelessness. These improvements were sustained during the 2‐month follow‐up period.^5^ Van der Spek et al.^9^ found in a RCT among cancer survivors that MCGP‐CS had larger treatment effects than CAU on personal meaning, goal orientedness, purpose in life, positive relations (all post intervention) and depressive symptoms (follow‐up). Compared to SGP, MCGP‐CS participants improved more on personal growth and environmental mastery (follow‐up). This RCT on MCP‐CS suggests that most positive postintervention effects fade away, but that some effects occur only several months later. Since cancer survivors often live for years with limitations in several areas of life, it is important to know whether MCGP‐CS's positive effects are maintained in the long‐term.

Several other types of existential interventions have been developed,^12^, ^13^, ^14^, ^15^, ^16^ and a few studies reported on the long‐term effects of these interventions. In four RCT's on experiential‐existential,^17^ cognitive‐existential,^13^ or supportive expressive group psychotherapy,^18^ participants improved over the 1‐year follow‐up period, but not more than after a non‐meaning‐focused type of group psychotherapy^16^, ^17^ or the care as usual condition.^12^, ^18^ In a RCT on cognitive existential couple therapy, couples did better after the existential therapy compared to care as usual, and these results were maintained during the 9‐month follow‐up period.^19^

The aim of this study is to investigate the long‐term follow‐up results of the RCT on the efficacy of MCGP‐CS by Van der Spek et al.^9^ Survivors' sense of personal meaning as well as psychological well‐being and posttraumatic growth were compared for MCGP‐CS, SGP, and CAU until 2 years after the intervention, both for all participants (intention‐to‐treat, ITT), and only for those who completed the intervention. Because many other things can also influence one's sense of meaning over the course of 2 years, in additional sensitivity analyses, psychological treatment and cancer recurrence during these 2 years were taken into account. Insight into the long‐term MCGP‐CS effects reveals whether this intervention supports survivors enduringly to experience a sense of meaning, well‐being, and growth, despite the limitations of having had cancer.

## 2 METHODS

### 2.1 Study design and population

This study is an extension of a multicenter RCT on the efficacy of MCGP‐CS compared to SGP and CAU with three follow‐up assessments: postintervention and at 3‐ and 6‐month follow‐ups. In the present study, assessments were added at 1‐ and 2‐year follow‐ups. To limit participant burden, only personal meaning, psychological well‐being, and posttraumatic growth were assessed at the long‐term follow‐ups. The study protocol and extension were approved by the Medical Ethics Committee of the Leiden University Medical Center and the trial was registered in the Netherlands Trial Register (NTR3571).

Details of the study procedure can be found in the previous report on the efficacy of MCGP‐CS.^8^ In brief, eligible patients were adult cancer survivors who were diagnosed in the last 5 years and who had completed treatment with curative intent. Participants had to have an expressed need for psychological care and at least one psychosocial complaint. Exclusion criteria were severe cognitive impairment, current psychiatric or psychological treatment elsewhere, and insufficient mastery of the Dutch language. Informed consent was obtained from all the individual participants included in the study.

### 2.2 Randomization and blinding

An independent researcher prepared a computer‐generated randomization table with random block sizes and made a list of sequentially numbered allocations. Participants were placed in a group, and when a consecutive group had 7 to 10 participants, the independent researcher allocated the group to a study arm.

### 2.3 Interventions

#### 2.3.1 Meaning‐centered group psychotherapy for cancer survivors

MCGP‐CS is a manualized intervention consisting of eight weekly 2‐hour sessions.^20^ The following themes were addressed: sources of meaning, meaning before and after cancer, past and future life stories as sources of meaning, participants' attitude toward life's limitations, creative sources of meaning, and experiential sources of meaning. In addition, important existential concepts played a role in MCGP‐CS, such as identity, existential guilt, isolation, and freedom.

#### 2.3.2 Supportive group psychotherapy

SGP is a manualized intervention that aims to help survivors cope better with the cancer‐related difficulties.^21^ Like MCGP‐CS, this intervention consists of eight weekly 2‐hour sessions. The themes addressed were as follows: need for support, communicating with health care providers, coping with medical tests, with family and friends, with vocational issues, body image, limitations in physical functioning, and coping with the future. Fidelity to both treatment protocols was ensured in several ways.^22^

### 2.4 Outcome measures

The primary outcome measure was personal meaning, measured as the total score of the Personal Meaning Profile (PMP).^23^, ^24^ The PMP has 39 items (*α* = .92) and five subscales: relation with God (*α* = .86), dedication to life (*α* = .89), fairness of life (*α* = .78), goal‐orientedness (*α* = .88), and relation with others (*α* = .85). All items were scored on a seven‐point Likert scale from 1 (not at all) to 7 (a great deal). The total and subscale scores were transformed to a scale of 0 to 100. Higher scores indicate a stronger sense of meaning. The PMP was validated in Dutch cancer patients and showed good internal consistency and construct validity.^24^

The 49‐item Dutch version of the Ryff's Scales of Psychological Well‐Being (SPWB) was used to measure psychological well‐being.^25^, ^26^ This measure has no total score. The original scale consists of six subscales: positive relations (*α* = .83), autonomy (*α* = .84), environmental mastery (*α* = .77), personal growth (*α* = .71), purpose in life (*α* = .79), and self‐acceptance (*α* = .81). In the Dutch version, two subscales of spiritual well‐being were added: inner strength (*α* = .75) and higher power (*α* = .91).^26^ Items were scored on a six‐point Likert scale ranging from 1 (strongly disagree) to 6 (strongly agree). Subscale scores were calculated as the mean item score and higher scores indicated greater well‐being.

Posttraumatic growth was measured using the total score of the Posttraumatic Growth Inventory.^27^, ^28^ This 21‐item measure (*α* = .91) has five subscales: relating to others (*α* = .85), new possibilities (*α* = .80), personal strength (*α* = .80), spiritual change (*α* = .70), and appreciation of life (α = .75). Items were rated from 0 (not at all) to 5 (very great degree). The total score was calculated as the sum of all items and a higher score represented more posttraumatic growth.

A study‐specific questionnaire was used to obtain sociodemographic characteristics. Clinical characteristics were retrieved from medical records. Uptake of psychological treatment was measured at baseline and all follow‐up assessments, except postintervention, using the items about psychiatric and psychological treatment of the Treatment Inventory of Costs in Patients with psychiatric disorders.^29^

### 2.5 Statistical methods

Linear mixed models (LMM) with fixed effects for study arm, time, and their two‐way interaction, as well as a random intercept for subjects, were used to investigate the differences in the course of the outcome measures over time in the three study arms. A correction was made for patient charactersitics if there were significant baseline differences across study arms. Also, analyses were corrected for the baseline scores of outcome measures in the case of significant differences between study arms at baseline. Results were reported on an ITT basis and for participants who attended six, seven, or all therapy sessions (completers).

If the course of an outcome measure differed significantly over time between the study arms, post hoc analyses were performed to assess which two groups differed significantly, using LMM, and between which points in time, using independent‐samples *t* tests. Post hoc analyses were corrected for multiple testing by Bonferroni's correction. Cohen's *d* effect sizes were calculated by dividing the difference in change since baseline between the study arms by the pooled SD, calculated at all separate follow‐up time points. Effect sizes of 0.2 were categorized as small, 0.5 as medium, and 0.8 as large.

As sensitivity analyses, all analyses were repeated without participants (a) who received psychological treatment during follow‐up and (b) who faced cancer recurrence during follow‐up. Analyses were performed in SPSS 24 and a two‐sided *P* value < .05 was considered to indicate statistical significance.

## 3 RESULTS

### 3.1 Study population

**Figure 1:**
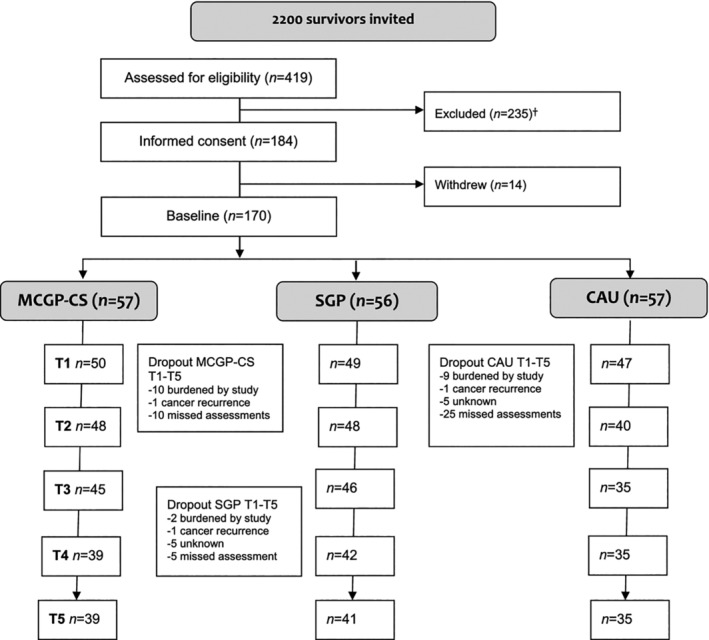
CONSORT diagram *Note:* MCGP‐CS, meaning‐centered group psychotherapy for cancer survivors; SGP, supportive group psychotherapy; CAU, care as usual; ^†^More details can be found in Van der Spek et al. (2017)

Basic information about the participant flow during the recruitment period and drop‐out in various phases of the study can be found in Figure 1 and is published in more detail elsewhere.^8^ Fifty‐seven survivors were randomly allocated to MCGP‐CS, 56 to SGP, and 57 to CAU. After 2 years, 39 (68%) of the MCGP‐CS participants, 41 (73%) of the SGP participants, and 35 (61%) of the CAU participants filled out the follow‐up questionnaire.

Sociodemographic and clinical participant characteristics are displayed in Table 1. Overall, most participants were female, in a relationship, and diagnosed with early stage breast cancer. Most completed the main cancer treatment about 1.5 year ago, and 80 (47%) still had hormonal therapy. In total, 29 (23%) participants received additional psychological treatment during follow‐up, 13 (8%) participants faced cancer recurrence, and 3 (2%) participants died.

**Table 1:** Participant characteristics

	MCGP (n = 57)		SGP (n = 56)		CAU (n = 57)		
	n	%	n	%	n	%	*P*
Age (*M*, *SD*, range)	59	11 (32‐81)	56	9 (41‐80)	57	10 (37‐83)	.34
Sex (female)	40	70	49	88	51	90	.012*
Marital status (single)	12	21	9	16	13	23	.65
Level of education							.16
Low	18	32	9	16	17	30	
Medium	20	35	25	45	15	26	
High	19	33	22	39	25	44	
Religion							.18
Christian	23	40	32	57	30	53	
No religion	34	60	24	43	27	47	
Past psychological treatment							.53
In the last year	12	21	11	20	7	13	
>1 year ago	21	37	21	37	17	31	
Never	24	42	24	43	31	56	
Psychological treatment during follow‐up	12	21	9	16	8	14	.61
Other negative life event in past 2 years (yes)	27	47	31	55	32	56	
Type of cancer							.071
Breast	30	53	40	71	42	74	
Colon	15	26	12	21	10	18	
Other	12	21	4	7	5	9	
Type of treatment							
Surgery	57	100	56	100	56	98	.37
Radiation	31	54	32	57	33	58	.92
Chemotherapy	26	46	34	61	36	63	.12
Hormonal therapy	22	39	28	50	30	53	.28
Months since last cancer treatment (median, range)	19	6‐58	16	5‐52	19	3‐55	.97
Cancer recurrence	3	5	5	9	5	10	.70
Mortality	1	2	2	4	0	0	

Abbreviations: CAU, care as usual; MCGP, meaning‐centered group psychotherapy; SGP, supportive group psychotherapy.

* *P* < .05.

### 3.2 Long‐term efficacy of MCGP‐CS

Significant differences between the three study arms in the course of the outcome measures over the period of 2‐year follow‐up were found on the primary outcome: personal meaning (PMP; *F*[10, 587] = 2.01, *P* = .030), and on the following secondary outcome measures: goal‐orientedness (PMP; *F*[10, 610] = 3.27, *P* < .001), positive relations (SPWB; *F*[10, 612] = 2.10, *P* = .022), and purpose in life (SPWB; *F*[10, 588] = 2.04, *P* = .028) (Table 2 and Table S1).

**Table 2:** Baseline, postintervention, and long‐term results of LMM analyzing treatment outcome^a^

				Short‐term			Long‐term							
	Baseline (T0)			1‐Week postintervention (T1)			1‐Year follow‐up (T4)			2‐Year follow‐up (T5)				
	MCGP, *M* (SD)	SGP, *M* (SD)	CAU, *M* (SD)	MCGP, *M* (SD)	SGP, *M* (SD)	CAU, *M* (SD)	MCGP, *M* (SD)	SGP, *M* (SD)	CAU, *M* (SD)	MCG, *M* (SD)	SGP, *M* (SD)	CAU, *M* (SD)	*P* ITT	*P* completers
PMP														
Total score	59 (16)	61 (13)	59 (12)	62 (16)	63 (13)	57 (14)	59 (15)	61 (14)	58 (13)	59 (17)	63 (12)	58 (11)	.030*	.011*
Goal‐orientedness	69 (20)	71 (17)	72 (17)	74 (20)	72 (16)	63 (23)	66 (17)	69 (18)	66 (18)	66 (20)	73 (17)	68 (16)	<.001**	<.001**
SPWB														
Positive relations^b^	4.1 (1.0)	4.5 (1.0)	4.5 (0.83)	4.4 (1.0)	4.7 (0.95)	4.4 (0.93)	4.4 (1.1)	4.6 (1.0)	4.5 (1.0)	4.4 (1.1)	4.6 (1.1)	4.3 (1.1)	.022*	.013*
Personal growth	4.2 (0.75)	4.4 (0.59)	4.3 (0.60)	4.4 (0.68)	4.4 (0.56)	4.3 (0.62)	4.4 (0.75)	4.3 (0.57)	4.3 (0.71)	4.2 (0.72)	4.3 (0.58)	4.3 (0.66)	.061	.029*
Purpose in life	4.1 (0.89)	4.3 (0.77)	4.4 (0.62)	4.4 (0.89)	4.3 (0.80)	4.3 (0.65)	4.2 (0.84)	4.3 (0.81)	4.4 (0.66)	4.3 (0.74)	4.3 (0.88)	4.3 (0.61)	.028*	.025*

Abbreviations: CAU, care as usual; ITT, intention‐to‐treat; MCGP, meaning‐centered group psychotherapy; PMP, Personal Meaning Profile; SGP, supportive group psychotherapy; SPWB, Scales of Psychological Well‐Being.

a Only significant results displayed.

b Corrected for baseline score.

*P < .05; **P < .005.

Post hoc LMM analyses with Bonferroni correction did not show a significant difference between two of the study arms in the course of personal meaning (PMP total score) and purpose in life (SPWB) from baseline to 2‐year follow‐up (Table 3). Stronger long‐term treatment effects of MCGP‐CS compared to CAU were found on goal‐orientedness (PMP; *F*[5, 392] = 4.97, *P* < .001) and positive relations (SPWB; *F*[5, 388] = 3.43, *P* = .025).

**Table 3:** Post hoc analyses: LMM analyzing difference between two study arms, and treatment effect postintervention and at long‐term follow‐up

	LMM analyses			Short‐term		Long‐term			
	From baseline to 2‐years follow‐up			Postintervention vs baseline		1‐Year follow‐up vs baseline		2‐Years follow‐up vs baseline	
	MCGP‐SGP	MCGP‐CAU	SGP‐CAU	MCGP‐SGP	MCGP‐CAU	MCGP‐SCP	MCGP‐CAU	MCGP‐SGP	MCGP‐CAU
	*P*	*P*	*P*	Cohen's *d*, *P*	Cohen's *d*, *P*	Cohen's *d*, *P*	Cohen's *d*, *P*	Cohen's *d*, *P*	Cohen's *d*, *P*
Intention‐to‐treat									
PMP									
Total score	1.00	.12	.17						
Goal‐orientedness	1.00	<.001**	.055		1.07, <.001**		0.12, 1.00		−0.04, 1.00
SPWB									
Positive relations^a^	1.00	.025*	.91		0.59, .008*		0.41, .21		0.82, .005*
Purpose in life	.22	.080	1.00						
Completers									
SPWB									
Personal growth	.020*	.28	1.00	0.65, .012*		0.94, .007*		0.34, .68	

Abbreviations: MCGP: meaning‐centered group psychotherapy; SGP: supportive group psychotherapy; CAU: care as usual; PMP: Personal Meaning Profile; SPWB: Scales of Psychological Well‐Being.

a LMM analyses are corrected for baseline score.

*P < .05; **P < .005.

Between‐group Cohen's *d* effect sizes of MCGP‐CS compared to CAU on goal‐orientedness (PMP) were large and significant (*d* = 1.07, *P* < .001) when comparing the posttreatment assessment with baseline assessment, but not on the longer‐term assessments. Effect sizes of MCGP‐CS compared to CAU on positive relations (SPWB) remained medium to large during the 2‐year follow‐up period and were significant when comparing the postintervention (T1; *d* = .59, *P* = .008) and 2‐year follow‐up (T5; *d* = .82, *P* = .005) assessment with baseline. 

### 3.3 Completers

For completers, the results were largely comparable (Table 2). Significant differences between study arms in the course of the outcome measure were found for personal meaning (PMP total score), goal‐orientedness (PMP), positive relations (SPWB), and purpose in life (SPWB). An additional significant result was found for personal growth (SPWB; *F*[10, 551] = 2.03, *P* = .029).

Post hoc analyses with Bonferroni correction did not reveal significant differences between two of the study arms for personal meaning (PMP total score) and purpose in life (SPWB). However, both MCGP‐CS participants (*F*[5, 368] = 5.22, *P* < .001) and SGP participants (*F*[5, 381] = 3.30, *P* = .030) scored significantly higher on goal‐orientedness (PMP) than CAU participants over the course of 2 years. Furthermore, MCGP‐CS participants scored significantly better on positive relations (SPWB) than CAU participants (*F*[5, 359] = 3.43, *P* = .025) and reported significantly more personal growth (SPWB) than SGP participants (*F*[5, 378] = 3.55, *P* = .020) (Table 3).

Compared with ITT analyses, the effect sizes of MCGP‐CS on goal‐orientedness (PMP) and positive relations (SPWB) were slightly larger. Effect sizes comparing the change in personal growth between baseline and the assessments postintervention (*d* = .65, *P* = .012), 3‐month follow‐up (*d* = .64, *P* = .017), and 1‐year follow‐up (*d* = .94, *P* = .007) were medium to large in favor of MCGP‐CS.

### 3.4 Sensitivity analyses

Both long‐term effects of MCGP‐CS on positive relations (SPWB; T5; *d* = .86, *P* = .010; compared to CAU) and personal growth (SPWB; T4; *d* = .76, *P* = .007; compared to SGP) remained significant when repeating the analyses without participants who received psychological treatment in the period from 4 weeks preceding the study to 2‐year follow‐up. In addition, at 2‐year follow‐up, MCGP‐CS participants reported more inner strength (SPWB) than CAU participants (*d* = .91, *P* = .007). No significant long‐term effects were found when repeating the analysis without participants who faced cancer recurrence during follow‐up.

## 4 DISCUSSION

In the present study, the effects of MCGP‐CS on personal meaning, psychological well‐being, and posttraumatic growth over a period of 2 years were compared to the effects of SGP and CAU. A previous study^8^ had shown that MCGP‐CS was effective in improving personal meaning, goal‐orientedness, positive relations, and purpose in life postintervention. The present study indicated that most of these effects fade 1 and 2 years after the intervention, including MCGP‐CS's positive effect on personal meaning. However, 2 years after MCGP‐CS occurred a medium to large positive effect on positive relations (compared to CAU). When analyzing completers only, MCGP‐CS had a large effect on personal growth 1 year later as well (compared to SGP). These long‐term results favored MCGP‐CS over the other conditions.

It is striking that none of the sources of meaning investigated in this study (eg, goal‐orientedness) were significantly affected by MCGP‐CS in the long‐term. The few long‐term effects that were identified all occurred on the measure of psychological well‐being (SPWB). It is possible that the SPWB is more sensitive for change than the measure that was used for personal meaning (PMP). An alternative explanation may be that the long‐term improvements in the area of psychological well‐being were not strong enough to be translated into an enhanced sense of meaning. MCGP‐CS's few long‐term effects on psychological well‐being were slightly stronger when analyzing completers only and without participants who received other psychological treatment during the follow‐up period. No long‐term effects were found when participants who faced cancer recurrence during follow‐up were left out. Further research is not only needed to validate these long‐term findings, but should also address the question how these long‐term intervention effects interact with other major events in life.

The long‐term results of MCGP‐CS seem to be in line with the results of previous studies on long‐term effects of existential interventions. Overall, these effects seem to be quite modest. However, while some other studies did not find significant differences between the long‐term effects of an existential intervention and a non‐meaning‐focused intervention^16^, ^17^ or care as usual,^13^, ^30^ the present study did find stronger improvement on some outcomes at long‐term follow‐up.

### 4.1 Clinical implications

MCGP‐CS is a useful addition to the current mental health care available in the oncology field. It is a brief intervention that is effective and cost‐effective.^8^, ^11^ Some of its effects linger on for 1 or even 2 years. Relatively easy adaptations could be made to stimulate stronger long‐term improvements of psychological well‐being and personal meaning. Meta‐analyses show that long‐term effects could be stimulated by more contact hours,^31^ possibly in the form of booster sessions.^32^ MCGP‐CS could also be extended with an online component, which can facilitate participants to remind and practice the skills they have learned.^33^

### 4.2 Study limitations

A strength of this study is its conservative ITT analyses with Bonferroni correction. The statistical methods decrease the chance of false positive findings, lending more credibility to the effects that were found. Yet, the possibility of chance findings could not be ruled out and the significant long‐term effects should be interpreted tentatively; especially, because some results appeared to be inconsistent. MCGP‐CS participants reported better positive relations (SPWB) at long term, but did not report that these relations became a stronger source of personal meaning (PMP) for them. Furthermore, personal growth was better after MCGP‐CS compared to SGP, but not compared to CAU, and only for completers of the intervention. In addition, there are no clear criteria for minimal important difference on the outcome measures used in this study, so it is unknown to what extent the significant differences are clinically meaningful.

Another limitation of this study is the omission of a measure of depressive symptoms at 1‐ and 2‐year follow‐up. The decision about which outcome measures to maintain at follow‐up was made before the short‐term results became available. However, when these results became available, an interesting finding was that symptoms of depression were significantly decreased after MCGP‐CS compared to CAU, but only at 6‐month follow‐up. Unfortunately, in the present long‐term study, we could not confirm whether this effect remained after 1 and 2 years.

## 5 CONCLUSIONS

In the 2 years after MCGP‐CS, there was a decay of the short‐term positive effect on personal meaning and most positive effects related to psychological well‐being. However, MCGP‐CS had a long‐term positive effect on positive relations with others and on survivors' sense of personal growth.

## CONFLICT OF INTEREST

K.H., B.L.W., and P.C. have nothing to disclose. N.v.d.S. and I.V.L. report grants form Dutch Cancer Society, during the conduct of the study. W.B. receives royalties from the sale of his manuals and textbook on MCP from Oxford University Press.

## ETHICS STATEMENT

All procedures performed in this study involving human participants were in accordance with the ethical standards of the institutional and national research committee (Medical Ethics Committee of the Leiden University Medical Center, P10.241) and with the 1964 Helsinki declaration and its later amendments.

## Supporting information


**Table S1**. Baseline, postintervention, and long‐term results of linear mixed models analyzing treatment outcome.

## Data Availability

The data that support the findings of this study are available on request from the corresponding author. The data are not publicly available due to privacy or ethical restrictions.
