# Supplementary material for: Long‐term efficacy of meaning‐centered group psychotherapy for cancer survivors: 2‐Year follow‐up results of a randomized controlled trial
Source: Psychooncology. 2020 Feb 6;29(4):711–8. doi: 10.1002/pon.5323 (PMC7199891; doi:10.1002/pon.5323)
Supplement: Supplementary file 1 — Table S1. Baseline, postintervention, and long‐term results of linear mixed models analyzing treatment outcome. [file PON-29-711-s001.docx]

Table 4 *Baseline, post-intervention and long-term results of linear mixed models analysing treatment outcome*

|  |  | | |  | Short-term | | |  | Long-term | | | | | | |  |  |
| --- | --- | --- | --- | --- | --- | --- | --- | --- | --- | --- | --- | --- | --- | --- | --- | --- | --- |
|  | Baseline (T0) | | |  | 1-week post-intervention (T1) | | |  | 1-year (T4) | | |  | 2-years (T5) | | |  |  |
|  | MCGP  *M (SD)* | SGP  *M (SD)* | CAU  *M* (*SD)* |  | MCGP  *M* (*SD)* | SGP  *M* (*SD)* | CAU  *M* (*SD)* |  | MCGP  *M* (*SD)* | SGP  *M* (*SD)* | CAU  *M* (*SD)* |  | MCGP  *M* (*SD)* | SGP  *M* (*SD)* | CAU  *M* (*SD)* | *p* ITT | *p com-pleters* |
| PMP |  |  |  |  |  |  |  |  |  |  |  |  |  |  |  |  |  |
| *Total score* | *59 (*16) | *61 (13)* | *59 (12)* |  | *62 (16)* | *63 (13)* | *57 (14)* |  | *59 (15)* | *61 (14)* | *58 (13)* |  | *59 (17)* | *63 (12)* | *58 (11)* | *.030** | *.011** |
| *Relation with God* | *32 (25)* | *39 (23)* | *30 (20)* |  | *36 (26)* | *40 (26)* | *29 (23)* |  | *34 (26)* | *41 (24)* | *32 (23)* |  | *32 (28)* | *42 (22)* | *31 (21)* | *.094* | *.065* |
| *Dedication to life* | *67 (19)* | *66 (16)* | *68 (15)* |  | *69 (18)* | *67 (15)* | *65 (17)* |  | *67 (17)* | *65 (16)* | *66 (17)* |  | *67 (19)* | *66 (15)* | *65 (15)* | *.43* | *.27* |
| *Fairness of life* | *56 (16)* | *55 (15)* | *55 (15)* |  | *60 (16)* | *59 (17)* | *54 (17)* |  | *58 (16)* | *58 (16)* | *58 (13)* |  | *60 (16)* | *60 (17)* | *57 (13)* | *.48* | *.50* |
| *Goal-orientedness* | *69 (20)* | *71 (17)* | *72 (17)* |  | *74 (20)* | *72 (16)* | *63 (23)* |  | *66 (17)* | *69 (18)* | *66 (18)* |  | *66 (20)* | *73 (17)* | *68 (16)* | *<.001** | *<.001** |
| *Relation* | *73 (24)* | *77 (22)* | *77 (19)* |  | *74 (24)* | *80 (20)* | *76 (20)* |  | *73 (24)* | *76 (22)* | *75 (22)* |  | *73 (25)* | *79 (20)* | *72 (21)* | *.36* | *.29* |
|  |  |  |  |  |  |  |  |  |  |  |  |  |  |  |  |  |  |
| SPWB |  |  |  |  |  |  |  |  |  |  |  |  |  |  |  |  |  |
| *Positive relations*† | *4.1 (1.0)* | *4.5 (1.0)* | *4.5 (.83)* |  | *4.4 (1.0)* | *4.7 (.95)* | *4.4 (.93)* |  | *4.4 (1.1)* | *4.6 (1.0)* | *4.5 (1.0)* |  | *4.4 (1.1)* | *4.6 (1.1)* | *4.3 (1.1)* | *.022** | *.013** |
| *Autonomy*‡ | *4.2 (.85)* | *4.3 (.83)* | *4.3 (.72)* |  | *4.4 (.80)* | *4.5 (.76)* | *4.3 (.76)* |  | *4.4 (.84)* | *4.4 (.85)* | *4.4 (.59)* |  | *4.4 (.80)* | *4.4 (.87)* | *4.4 (.70)* | *.51* | *.62* |
| *Environmental mastery* | *4.2 (.74)* | *4.3 (.71)* | *4.4 (.72)* |  | *4.5 (.75)* | *4.3 (.79)* | *4.4 (.67)* |  | *4.3 (.78)* | *4.3 (.76)* | *4.4 (.71)* |  | *4.4 (.82)* | *4.3 (.83)* | *4.4 (.72)* | *.053* | *.074* |
| *Personal growth* | *4.2 (.75)* | *4.4 (.59)* | *4.3 (.60)* |  | *4.4 (.68)* | *4.4 (.56)* | *4.3 (.62)* |  | *4.4 (.75)* | *4.3 (.57)* | *4.3 (.71)* |  | *4.2 (.72)* | *4.3 (.58)* | *4.3 (.66)* | *.061* | *.029** |
| *Purpose in life* | *4.1 (.89)* | *4.3 (.77)* | *4.4 (.62)* |  | *4.4 (.89)* | *4.3 (.80)* | *4.3 (.65)* |  | *4.2 (.84)* | *4.3 (.81)* | *4.4 (.66)* |  | *4.3 (.74)* | *4.3 (.88)* | *4.3 (.61)* | *.028** | *.025** |
| *Self-acceptance* | *4.0 (.87)* | *4.2 (.80)* | *4.3 (.64)* |  | *4.2 (.82)* | *4.3 (.83)* | *4.3 (.63)* |  | *4.2 (.77)* | *4.1 (1.0)* | *4.3 (.72)* |  | *4.2 (.84)* | *4.1 (.85)* | *4.3 (.66)* | *.53* | *.64* |
| *Inner strength* | *4.0 (.69)* | *4.1 (.80)* | *4.1 (.66)* |  | *4.3 (.80)* | *4.2 (.74)* | *4.1 (.78)* |  | *4.2 (.90)* | *4.3 (.84)* | *4.2 (.65)* |  | *4.3 (.78)* | *4.3 (.71)* | *4.0 (.72)* | *.12* | *.064* |
| *Higher power* | *2.8 (1.4)* | *3.1 (1.3)* | *2.8 (1.2)* |  | *3.0 (1.3)* | *3.2 (1.3)* | *2.7 (1.2)* |  | *3.0 (1.4)* | *3.2 (1.2)* | *2.6 (1.1)* |  | *2.9 (1.4)* | *3.1 (1.2)* | *2.9 (1.2)* | *.28* | *.055* |
|  |  |  |  |  |  |  |  |  |  |  |  |  |  |  |  |  |  |
| PTGI | 42 (21) | 48 (17) | 46 (18) |  | 48 (20) | 53 (17) | 49 (19) |  | 44 (24) | 50 (17) | 50 (21) |  | 45 (21) | 50 (19) | 47 (20) | *.77* | *.46* |

*Note.* MCGP: meaning-centered group psychotherapy; SGP: supportive group psychotherapy; CAU: care as usual; PMP: Personal Meaning Profile; SPWB: Scales of Psychological Well-Being; PTGI: posttraumatic growth inventory; ITT: intention-to-treat.

This table is the complete version of Table 2.

†Corrected for baseline scores.

‡Corrected for sex.

**p*<.05
